# Clinical pharmacist led hospital-wide direct oral anticoagulant stewardship program

**DOI:** 10.1186/s13584-019-0285-9

**Published:** 2019-02-01

**Authors:** Amichai Perlman, Ehud Horwitz, Bruria Hirsh-Raccah, Gefen Aldouby-Bier, Tamar Fisher Negev, Sarit Hochberg-Klein, Yosef Kalish, Mordechai Muszkat

**Affiliations:** 1 Department of Pharmacy, Hadassah Hebrew University Medical Center, Jerusalem, Israel; 2 Department of Medicine, Hadassah Hebrew University Medical Center, Mt Scopus, Jerusalem, Israel; 3 Department of Cardiology, Hadassah Hebrew University Medical Center, Jerusalem, Israel; 4 Department of Oncology, Hadassah Hebrew University Medical Center, Jerusalem, Israel; 5 Department of Orthopedics, Hadassah Hebrew University Medical Center, Jerusalem, Israel; 6 Department of Hematology, Hadassah-Hebrew University Medical Center, Jerusalem, Israel

**Keywords:** Direct oral anticoagulants, Clinical pharmacist, Potentially inappropriate prescribing, Drug related problems, Drug safety

## Abstract

**Introduction:**

In the past decade, direct-acting oral anticoagulants (DOAC) have been introduced to medical practice for several indications, with a wide range of dosing regimens. As both over- and under-dosing might lead to life-threatening events, development of methods promoting safe and effective utilization of these agents is imperative. The Hadassah Clinical Pharmacy team initiated a hospital-wide program, for monitoring and promoting safe and effective prescription of DOAC during hospitalization. This study describes the types of drug related problems addressed and the program’s performance in terms of consultation rates and physician acceptance.

**Methods:**

Electronic medical records throughout the hospital were screened for DOAC orders. All DOAC orders were assessed by a clinical pharmacist for potentially-inappropriate prescribing. When potentially-inappropriate prescribing or a drug-related problem was identified, the clinical pharmacist provided consultation on management options. In specific cases, additional guidance was provided by coagulation and pharmacology specialists. Data on patient characteristics, clinical pharmacist consultations, and physician response was retrospectively retrieved for the first six months of 2017. Characteristics of patients with and without consultations were compared, consultations were categorized by the recommended management of the drug related problem, and physician acceptance rates were evaluated by category.

**Results:**

During the evaluated period, 585 patients with DOAC orders were identified. Patients were evenly distributed by gender, and age averaged 78 years. Most patients received apixaban (75%) followed by rivaroxaban (14%) and dabigatran (11%), and most (63%) received “reduced dose” regimens. Clinical pharmacists provided 258 consultations for 210 patients, regarding anticoagulation management, such that more than one in three patients on DOAC had potentially inappropriate prescribing or drug related problems. Consultations included alerts regarding potentially inappropriate DOAC doses and recommendations to increase (29%) or decrease (5%) the dose, potentially inappropriate concomitant antiplatelet agents (20%), need for DOAC level monitoring (23%), and alerts regarding other drug related problems (23%). More than 70% of recommendations were accepted by the attending physician.

**Conclusion:**

Due to the complexity of DOAC management, potentially-inappropriate prescribing and drug related problems are common. Multidisciplinary collaborative projects including review and consultation by clinical pharmacists are an effective method of improving management of patients on DOAC.

**Trial registration:**

Retrospectively registered at clinicaltrials.gov, NCT03527615.

## Introduction

The introduction of direct acting oral anticoagulants (DOAC) into clinical practice over the past decade has dramatically changed the field of stroke and thrombosis prevention and treatment. These medications include the direct thrombin-inhibitor dabigatran, and the direct factor Xa inhibitors rivaroxaban, apixaban, edoxaban, and betrixaban. The DOAC have been found to be similarly effective in preventing thromboembolic events when compared to traditional anticoagulant treatment in a variety of health conditions, and tended to have a lower risk of major bleeding, particularly intracranial hemorrhage [1, 2]. The uptake of DOAC in clinical practice has been rapid, and has already surpassed the use of traditional anticoagulants, especially when used for the prevention of stroke and thromboembolism in patients with atrial fibrillation (AF) [3, 4].

Perhaps the main consideration driving the rapid adoption of these medications into clinical practice is their relative simplicity of use compared to traditional older anticoagulant agents. The most commonly used older anticoagulants are vitamin-K antagonists (VKA), such a warfarin, and low-molecular-weight heparins (LMWH), such as enoxaparin. VKA have an unpredictable response, requiring individualized dosing, continuous and frequent laboratory monitoring, and special care and awareness regarding drug-drug and food-drug interactions. LMWH have a more predictable dose-response profile, however they require once to twice daily injections, and their use has not been extensively evaluated in indications requiring long-term use. In contrast, DOAC have been evaluated in studies utilizing nearly uniform doses, and do not require laboratory monitoring nor multiple injections. DOAC have thus far been formally evaluated and approved for: (1) treatment and prevention of stroke and thromboembolic events in patients with atrial fibrillation, (2) treatment and prevention of deep vein thrombosis (DVT) or pulmonary embolism (PE), and (3) prevention of DVT/PE following total knee replacement (TKR) or total hip replacement (THR).

Despite their relative safety profile and seeming simplicity of use, the adoption of DOAC in practice has introduced several significant challenges. Each of the DOAC has been tested using a range of indication-dependent doses, leading to a wide range of doses approved for each DOAC, varying based on indication, treatment duration, renal function, patient weight and concomitant medications. The introduction of several DOAC within a short time period further contributed to the complexity related to DOAC utilization. Consequently, errors, misunderstandings, confusion and inappropriate DOAC prescribing and use are common,^7,8^ and are of considerable significance in light of the continuously-expanding utilization of DOAC in clinical practice.

The optimal utilization of DOAC is a clinical imperative, as anticoagulant therapy is a double-edged sword, with significant associated risk, requiring a careful watchful balance of the expected benefit in reduction of thromboemoblic events with the increased risk of bleeding. Recent studies show that anticoagulation-associated hemorrhage is among the most common causes of drug-related emergency department visits [5], while inappropriately reduced DOAC doses might be associated with increased risk of stroke and thromboembolism [6].

The effectiveness of medication management by clinical pharmacists has been documented in a wide variety of areas, including the treatment of diabetes, blood pressure, and dyslipidemia, and management of polypharmacy, medication safety, and adherence [7]. Studies have likewise demonstrated improved care with the addition of clinical pharmacy services in the hospital setting, including antimicrobial stewardship, therapeutic drug monitoring, medication reconciliation, and anticoagulation use [8]. Similar results have been reported with the introduction of clinical pharmacy services to the hospital setting in Israel [9–11]. The success of these services have led the The Agency for Healthcare Research and Quality to list “Use of clinical pharmacists to reduce adverse drug events” among “The Top Patient Safety Strategies That Can Be Encouraged for Adoption Now” [12], and a recent systematic review concluded that pharmacist-managed anticoagulation attained better quality of anticoagulation control, lower bleeding and thromboembolic events, and lower health care utilization [13].

In this study, we describe and evaluate an initiative to improve DOAC utilization via programmatic review by clinical pharmacists in collaboration with coagulation specialists, including two hematologists and a clinical pharmacologist, in the Hadassah University Hospitals in Jerusalem. The study describes the types of drug related problems addressed and assesses its performance in terms of consultation rates and physician acceptance.

## Methods

### Settings and population

This study is a retrospective evaluation of an initiative to improve DOAC utilization in hospitalized patients. The study was performed at the Hadassah Hebrew University Medical Center, which consists of two hospitals: an 800-bed tertiary care hospital at Hadassah Ein-Kerem, and a 350-bed community hospital at Hadassah Mt. Scopus. Both hospitals operate in Jerusalem, Israel, and share institutional resources, including an electronic health record system.

The initiative included all patients prescribed a DOAC during hospitalization, regardless of indication, as the aim was to reduce all potentially inappropriate prescribing of these high-risk medications. As identifying DOAC prescriptions required a computerized query of electronic health records in all departments. The only patients excluded were patients hospitalized in departments not using the institution electronic health records – i.e. the emergency rooms and intensive care units.

The retrospective evaluation and retrieval of data from patient records reviewed in this program was approved by the institutional Helsinki committee (365–15-HMO).

### Intervention

The initiative to improve DOAC utilization via programmatic review by clinical pharmacists was developed in collaboration with clinical pharmacology and hematology specialists. The clinical pharmacists were all licensed pharmacists with advanced degrees in clinical pharmacy, whose clinical training included education and experience in medication therapy management in general, and with anticoagulant therapy specifically.

The initiative was designed following an extensive literature review, including the detailed evaluation of phase III DOAC studies (as well as analyses of their safety in specific sub-populations) [14, 15], review of regulatory and medical society guidelines of DOAC use, and review of the literature on clinical pharmacist led interventions for medication management in general, and management of anticoagulation specifically [8, 13].

All institutional electronic medical records were screened for physician DOAC orders, using an automated query, at least twice-weekly. All DOAC orders were thoroughly reviewed by a certified clinical pharmacist. The clinical pharmacist review included assessment of appropriateness of DOAC indication, selection, dose and regimen, taking into account patient age, weight, estimated renal function, potential drug-drug and drug-disease interactions, and other factors with potential impact on clinical management. A national medical database linking ambulatory and hospital data was accessed for obtaining additional clinical information, as needed.

When potentially inappropriate prescribing or drug-related problems were identified, the clinical pharmacist alerted the attending physician and provided consultation on possible management options. Potentially inappropriate prescribing was defined as prescribing of DOAC which was not in accordance to the guidance of regulatory and medical authorities, including the Ministry of Health approved prescribing information for apixaban (Eliquis), dabigatran (Pradaxa), and rivaroxaban (Xarelto), [16–18] as well as medical society guidelines (the European Society of Cardiology guidelines for managing AF, and the European Heart Rate Association’s guide to DOACs) [19–21]. These included potentially inappropriate doses, absence of approved indication, presence of a contra-indication, and potential drug-drug and drug-disease interaction. Other drug-related problems included medication reconciliation (i.e. discrepancy in the identity of the DOAC agent or dose prescribed prior and during hospitalization), concern regarding patient adherence or medication accessibility, difficulty or inability to administer the medication orally, and the presence of other unique patient characteristics requiring clinical consideration (such as use of DOAC in morbidly-obese patients, or those of extremely advanced age or with severely-impaired renal function). Consultations and management options included suggestions for changes in doses, changes in concomitant medication, and laboratory monitoring. Laboratory monitoring was considered appropriate per institutional policy in unique patients likely not represented in phase-III trials, when they had characteristics likely to influence DOAC’s pharmacokinetics and pharmacodynamics. This approach to DOAC monitoring is similar to that endorsed by the European Heart Rate Association’s guide to DOACs [21].

The institution’s coagulation specialists, including two hematologists and a clinical pharmacologist, provided guidance for general management strategies, as well as consultation and support in specific cases, as required. Consultations and alerts were recorded in the electronic medical record. Performance measures for program activity, including follow-up of acceptance and implementation of consults, were collected and summarized for the first six months of 2017.

### Variables

Data summarized included characteristics of patients with DOAC, and characteristics of interventions by clinical pharmacists. Patient characteristics included age, gender, and DOAC dose. To facilitate retrospective evaluation of clinical pharmacist consultations, they were broadly categorized by the type of recommendation given into the following categories: monitor DOAC levels, increase dose, decrease dose, stop concomitant antiplatelet, and others. In addition, the acceptance of the recommendation by the physician was recorded.

A larger range of characteristics was retrieved for a subset of 289 patients from the internal medicine department to compare characteristics of patients with and without clinical pharmacist consultation. Characteristics compared included age, gender, weight, serum creatinine, DOAC dose, concomitant antiplatelets, and concomitant medications which inhibit CYP3A4 and Pgp (P-glycoprotein) and are therefore expected to interact with DOAC (i.e. amiodarone, dronedarone, fluconazole, verapamil, diltiazem, cyclosporine, tacrolimus, etc.).

### Statistics

Continuous variables were expressed as means with standard deviation (SD). Categorical variables were expressed as frequencies and percentages. Characteristics of patients with and without consultation were compared using univariate tests. Significance between groups was assessed by the Chi square test or Fisher’s exact test for categorical variables, and the t-test or Mann-Whitney U test for continuous variables. Normality of continuous variables was evaluated by visual inspection of density plots, Q-Q plots and Shapiro-Wilk test for normality. Analyses were conducted using R version 3.4.3 [22].

## Results

During the period evaluated, 585 patients with orders for DOAC were identified. Patients’ age averaged 78 years (SD 10), with gender approximately evenly distributed (48% female). Most DOAC orders were for apixaban (75%) followed by rivaroxaban (14%) and dabigatran (11%). The majority of orders (63%) were for a reduced DOAC dose (being lower than the maximal dose per indication, as listed in Table 1).

**Table 1:** DOAC - Approved Indications and Dosing

	Apixaban (18)	Rivaroxaban (17)	Dabigatran (16)
Prevention of VTE in patients undergoing elective HRS or KRS.	2.5 mg BID for 32-38d (HRS) or 10-14d (KRS)	10 mg OD for 5wk (HRS) or 2wk (KRS)	220 mg OD for 28-35d (HRS) or 10d (KRS); Reduce to 150 mg OD if CrCl = 30-50 ml/min OR age ≥ 75 yr. OR concomitant amiodarone, quinidine or verapamil
Treatment of DVT and PE, and prevention of recurrent DVT and PE in adults.	10 mg BID for 1wk, then 5 mg BID for up to 6mo, then 2.5 mg BID for prevention	15 mg BID for 3wk, then 20 mg OD	150 mg BID
Prevention of atherothrombotic events after an ACS	NI	2.5 mg BID for up to 12mo	NI
Prevention of stroke and systemic embolism in patients with NVAF.			
Full dose	5 mg BID	20 mg OD	150 mg BID
Reduced dose	2.5 mg BID if CrCl< 30 ml/min, OR if 2 of 3: Age > 80 Weight < 60 SCr > 135 (avoid if CrCl< 15)	15 mg OD if CrCl = 15–50 (avoid if CrCl< 15)	110 mg BID if age > 80 OR concomitant verapamil (avoid if CrCl< 30)

*ACS* acute coronary syndromes, *BID* twice daily, *CrCl* creatinine clearance, *DVT* deep vein thrombosis, *HRS* hip replacement surgery, *KRS* knee replacement surgery, *NI* not indicated, *NVAF* non-valvular atrial fibrillation, *OD* once daily, *PE* pulmonary emboli, *VTE* venous thromboembolism

During the 6-month study period, clinical pharmacists provided a total of 258 alerts and consultations regarding anticoagulation management, for 210 patients (36% of all patients reviewed). These included recommendations to monitor DOAC and anti-Xa levels (23% of all recommendations), increase (29%) or decrease (5%) DOAC dose, discontinue concomitant antiplatelet drug (20%), and other recommendations pertaining to DOAC therapy management (23%), such as need for acid-suppressing medications and anticoagulant duplication.

**Fig. 1:**
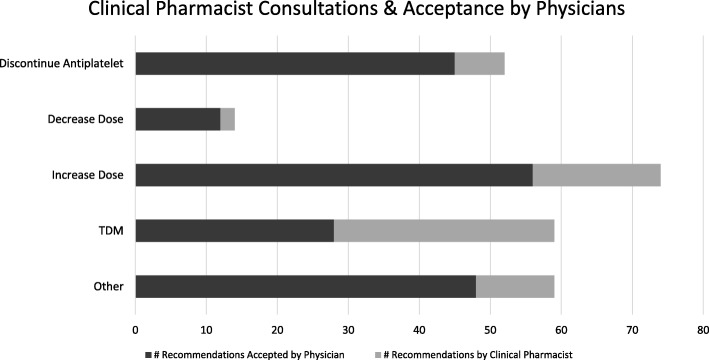
Figure presents number of recommendations made by clinical pharmacists, and number of recommendations accepted by the attending physician, during the study period according to five categories: recommendations to consider discontinuing concomitant antiplatelet therapy, decrease dose, increase dose, monitoring the plasma level of the anticoagulant (TDM), and “other” recommendations

Of the 258 consults provided, 189 were accepted and implemented in clinical management, for an overall physician acceptance rate of 73%. Physicians’ acceptance rate was highest for recommendations to stop concomitant antiplatelet agents (87%) and lowest for recommendations for drug level monitoring (47%) (Fig. 1).

In the subset of the 289 internal medicine patients, only younger age and antiplatelet use were found to be significantly associated with need for clinical pharmacist consultation. Other patient characteristics investigated were not found to be associated with potentially inappropriate prescribing or drug-related problems requiring clinical pharmacist consultation, including: gender, weight, serum creatinine, use of full dose of DOAC, or use of concomitant CYP/Pgp inhibitors (Table 2).

**Table 2:** Characteristics of Subset of Internal Medicine Patients with and without DOAC Consultation

	+ Consultation *n* = 53	- Consultation *n* = 236	*p*-value
Age in years	77 ± 10	81 ± 10	0.02^a^
Female	19 (36%)	122 (52%)	0.09^b^
Weight in kg	76 ± 13	70 ± 49	0.05^c^
Serum Creatinine in μmol/l	102 ± 41	115 ± 62	0.19^a^
Full dose	15 (28%)	73 (31%)	0.08^d^
Antiplatelet	26 (49%)	25 (11%)	< 0.001^b^
CYP/Pgp inhibitors	12 (23%)	65 (28%)	0.57^b^

^a^Mann-Whitney U test

^b^Chi square test

^c^t-test

^d^Fisher’s exact test for categorical variables

## Discussion

In this study we found a high rate of potentially inappropriate prescribing and drug-related problems in patients hospitalized with DOAC. Medication orders for DOAC led to alert and consultation by clinical pharmacists in one of every three patients prescribed DOAC. Patients with DOAC orders requiring clinical pharmacist consult were, on average, younger and more likely to be receiving concomitant antiplatelet therapy, than those with medication orders deemed appropriate. Over 70% of these consults were accepted by the treating physicians.

The high prevalence of potentially inappropriately prescribing of DOAC in hospitalized patients revealed in the current project is concerning. The incidence of serious and fatal ADRs in hospital is high [23], and anticoagulants have been reported to be a major, and probably primary, contributor to serious adverse drug events [5]. Our findings should prompt efforts and strategies to improve prescribing of these high risk medications.

The high acceptance rates of clinical pharmacist alerts and consults in this study (73%) contrast dramatically with the very low rates reported with computerized methods to reduce medication error and drug-related problems. Studies of computerized alerts have reported much lower acceptance rates, with some studies reporting acceptance rate as low as 4–5% [24, 25]. Similar high acceptance rates of clinical pharmacist alerts and consults have been reported in other medication management studies. Studies have reported a 60% acceptance rate for clinical pharmacist recommendations in a university hospital geriatric ward [26], 85% acceptance of clinical pharmacist recommendations in internal medicine [27], and 80% acceptance of recommendations by a hospital pharmacy anticoagulation management program [28]. These higher acceptance rates likely demonstrate both the greater clinical relevance of the recommendations, as well as the effectiveness of professional human interaction and collaboration.

While the bleeding risk associated with the use of anticoagulation is intuitive, and readily apparent, over-cautious use of DOAC might also pose significant health hazards. Patients with an indication for DOAC generally face a significantly increased risk of thromboembolic events, and under-treatment, or use of lower doses than required, might result in increased morbidity and mortality. The danger of under-dosing of DOAC has been suggested in recent studies reporting higher risk of stroke in patients receiving lower doses [6, 29, 30], and a number of studies have documented a tendency to prefer lower doses of DOAC [29–32]. The tendency to prescribe lower doses of DOAC is noticeable in our cohort in both the high rate of low-dose utilization (63%), and the higher rate of clinical pharmacist recommendations to increase dose (29%) compared to the rate of consultations to reduce dose (5%). In addition, as according to therapeutic and prescribing guidelines the reduction of DOAC dose is more likely to be appropriate with increasing age, as well as with age associated decline in renal function, we found clinical pharmacist consultations were significantly more likely to be given to younger patients.

The use of antiplatelet agents in patients using anticoagulants is associated with a significant increase in the risk of major bleeding [33–35]. Therapeutic guidelines strongly discourage the use of antiplatelets in patients receiving anticoagulants, and encourage DOAC monotherapy even in patients with indications traditionally treated with antiplatelets, such as those with a history of coronary artery disease [20]. In this study, one in five clinical pharmacist consultations were dedicated to the discontinuation of concomitant antiplatelet use. This is also in line with our finding that patients receiving clinical pharmacist consultation were significantly more likely to have been receiving concomitant antiplatelet therapy.

Of the consultations provided, advice encouraging DOAC concentration monitoring was accepted least often (46%). DOAC were studied, approved, and marketed for use without a need of continuous and frequent laboratory monitoring, and there is limited data and evidence to guide the clinical interpretation of DOAC concentration monitoring. It is therefore reasonable for physicians to be reluctant to conduct such testing. However, there is increasing recognition that monitoring may be helpful in certain clinical settings [36–38]. These classically include cases of overdose or requiring emergency surgical procedures.

In our institution, laboratory monitoring is often also considered in unique patients likely not represented in phase-III trials, when they have characteristics likely to influence DOAC’s pharmacokinetics and pharmacodynamics. These include patients with very advanced age (patient ages in this study ranged up to 103 years), morbidly-obese patients, those with severe renal failure, and those with potentially-interacting medications. Studies have suggested a potential for differences in response among morbidly-obese and post-bariatric patients patients [39–41]. Likewise, while apixaban and rivaroxaban are approved for use in renal failure with CrCl> 15 ml/min, DOAC levels and risk of bleeding increase with the reduction in renal function, and there is a paucity of data regarding DOAC use in severe renal failure (CrCl< 30 ml/min) [14].

Our study presents the feasibility and effectiveness of a collaborative multidisciplinary initiative, involving clinical pharmacists, hematologists, and clinical pharmacologists, to improve prescribing of DOAC. Inappropriately high or low doses of DOAC, concomitant use of antiplatelet medication, and DOAC use in high risk patients (such as those with suspected drug-drug interactions, extremes of age, weight, or renal function), have been clearly associated with increased risks for bleeding and thromboembolic events. In our study, clinical pharmacists provided continuous monitoring of anticoagulation prescribing, and provided alerts and consultation on a wide range of medication related problems. This activity was met by a high level of acceptance by the physicians, thereby reducing potentially inappropriate prescribing of DOAC and improving patient safety.

This initiative and its evaluation have several strengths and limitations. The study provides data on DOAC prescribing in “real world” hospitalized patients in Israel, across two hospitals, in a wide variety of departments, and highlights some important challenges in prescribing these high-risk medications. Additionally, the study provides an overview of a project aimed at reducing potentially inappropriate prescribing and drug-related problems and evaluates its effectiveness in terms of acceptance by treating physicians. However, the study has a number of limitations. The study did not include an evaluation of the impact of this project on clinical outcomes (such as the rate of bleeding or thromboembolic complications). The project was based on computerized query of physician orders for DOAC and therefore did not include a few departments not using this system (the intensive care units and emergency departments). Additionally, the intervention included both patients who initiated therapy with DOACs in the community as well as those initiating therapy in the hospital, and we could not retrospectively evaluate possible differences between these groups. Lastly, the evaluation of patient characteristics linked to clinical pharmacist consultation was not performed on the full cohort but only on a subset of patients from the internal medicine department.

### Clinical policy implications

Our study has significant implications for public health policy. The high prevalence of inappropriate prescribing and drug-related problems with DOAC identified in this study highlight a pressing need to promote strategies to reduce risk with these medications. The effectiveness of medication management by clinical pharmacists has been previously documented in a wide variety of areas and settings [7–11]. This study demonstrates the feasibility of multidisciplinary collaborative projects involving clinical pharmacists, hematologists, and clinical pharmacologists, in promoting safe and effective use of anticoagulants in the hospital setting. Additional studies are needed to measure the impact of such projects on clinical outcomes.

As anticoagulants are one of the leading culprits of drug related adverse events [5], additional measures should be considered to ensure safe utilization of these medications. Due to technical and resource constraints, our anticoagulant stewardship program did not cover departments not using the institutional electronic medical record system for patient management and provided only bi-weekly review of prescriptions. To maximize patient safety, all medication orders, especially of high-risk medications, in all departments, require review by specialized personnel in real time, prior to medication use. This standard is endorsed and widely applied in the U.S. [42], where nearly all pharmacists hold advanced degrees (PharmD) and receive significant advanced clinical training, and where many logistic aspects of pharmacy management are performed by “pharmacy technicians”. Such intensive processes are not currently attainable in Israel, as there is a limited number of highly trained clinical pharmacists, and the logistic aspects of hospital pharmacy services occupy nearly all the hospital pharmacists’ time. As the population in Israel is aging, and the number and complexity of medication management continue to increase, there is a growing need for specially trained personnel to monitor and consult on medication management. Shifting the focus of pharmacist activities in the hospital, and revising in the training of pharmacists in Israel, should be considered to address this need.

Advanced clinical pharmacy training has been provided by the Hebrew University of Jerusalem for the past two decades on a very small scale. There are currently an estimated 150 clinical pharmacists practicing in Israel, mostly trained in this program. Recently the Ben-Gurion University has opened an advanced degree in clinical pharmacy as well. These programs would need to grow significantly to train enough clinical pharmacists to provide professional monitoring and consultation of medication related issues throughout the Israeli health-system. To achieve this goal, universities should consider transitioning pharmacist training in Israel to the U.S. model, where pharmacy training and licensing exists only as an advance degree incorporating significant clinical training. However, such a transition must be accompanied by concrete steps by the Ministry of Health to regulate and define licensing and staffing standards, as well as requires that the Ministry of Finance provide the necessary funding to facilitate the wide-spread incorporation of clinical pharmacy services in the Israeli health system.

In addition, automated computerized methods should be developed to increase safe prescribing of medications in general, and DOACs specifically. While studies of clinical decision support systems have reported high rates of “alert fatigue” poor uptake by clinicians [24, 25], it is likely that electronic prescription processes can be designed to reduce some types of prescription errors, via more implicit guidance – such as by offering a range of standard dosing suggestions as part of the prescription process, or by passively highlighting patients’ bleeding risk. Moreover, while computerized systems may not be able to replace professional human interactions, electronic algorithms can likely be used to augment the monitoring of high-risk medications, such as by highlighting “outlier” prescriptions or via rule base systems which can be tailored by the user to be relevant to specific practice settings, thereby reducing false alarms.

Many of the drug related problems identified in this study existed prior to hospitalization, and chronic medication management is primarily the responsibility of primary care physicians. It is therefore clear that improving the safety of medications also necessitates the development and provision of programmatic, focused, and continued education to primary physicians in this area. There is a shortage of physicians and nurses in the Israel health system, and there are only a select few with advanced training in clinical pharmacology. Increasing the number of clinical pharmacists in Israel, could therefore also provide the professional personnel needed to develop and provide guidance and education on medication management and safety in the primary care setting.

## Conclusion

Due to the complexity of DOAC management, potentially-inappropriate prescribing and drug related problems are common. The high prevalence of inappropriate prescribing and drug-related problems with DOAC identified in this study highlight a pressing need to promote strategies to reduce risk with these medications. Multidisciplinary collaborative projects including review and consultation by clinical pharmacists with the involvement of hematologists and clinical pharmacologists are an effective method of improving management of patients on DOAC. Implementation of similar projects on a country-level scale would require significant changes in the training of pharmacists in Israel, and in the scope of hospital-pharmacy activity in the Israeli health system. Additional studies are needed to measure the impact of such projects on clinical outcomes.

## Abbreviations

ACS: Acute coronary syndromes
AF: Atrial fibrillation
BID: Twice daily
CrCl: Creatinine clearance
DOAC: Direct oral anticoagulants
DVT: Deep vein thrombosis
HRS: Hip replacement surgery
Kg: Kilogram
KRS: Knee replacement surgery
NI: Not indicated
NVAF: Non-valvular atrial fibrillation
OD: Once daily
PE: Pulmonary emboli
Pgp: P-glycoprotein
TDM: Therapeutic drug monitoring
VTE: Venous thromboembolism
